# Fluocinolone acetonide 0.2 µg/day intravitreal implant in non-infectious uveitis affecting the posterior segment: EU expert user panel consensus-based clinical recommendations

**DOI:** 10.1186/s12348-024-00402-4

**Published:** 2024-05-30

**Authors:** Uwe Pleyer, Carlos Pavesio, Elisabetta Miserocchi, Carsten Heinz, Helen Devonport, Víctor Llorenç, Tomás Burke, Vanda Nogueira, Laurent Kodjikian, Bahram Bodaghi

**Affiliations:** 1 Charité – Universitätsmedizin Berlin, Augustenburger Platz 1, 13353 Berlin, Germany; 2 Department of Ophthalmology at Moorfields Eye Hospital, London, UK; 3 Department of Ophthalmology, Ocular Immunology and Uveitis Service, University Vita-Salute, IRCCS San Raffaele Scientific Institute, Milan, Italy; 4 Department of Ophthalmology at St, Franziskus Hospital Muenster, Münster, Germany; 5 Department of Ophthalmology, University Duisburg-Essen, Essen, Germany; 6 Bradford Teaching Hospitals NHS Foundation Trust, Bradford, West Yorkshire UK; 7 Clínic Hospital of Barcelona, Barcelona, Spain; 8 August Pi I Sunyer Biomedical Research Institute (IDIBAPS), Barcelona, Spain; 9 Department of Ophthalmology, Mater Misericordiae University Hospital, Dublin, Ireland; 10 Instituto de Oftalmologia Dr. Gama Pinto, Lisbon, Portugal; 11 Service d’Ophtalmologie, Hôpital Universitaire de La Croix-Rousse, Hospices Civils de Lyon, 69004 Lyon, France; 12 UMR5510 MATEIS, CNRS, INSA Lyon, Université Lyon 1, 69100 Villeurbanne, France; 13 Department of Ophthalmology, Pitié-Salpêtrière Hospital, Paris, France

**Keywords:** Fluocinolone intravitreal implant, Yutiq, ILUVIEN, Intraocular inflammation, Non-infectious uveitis, Posterior segment

## Abstract

**Background:**

Non-infectious uveitis affecting the posterior segment of the eye (NIU-PS) is an inflammatory disease, which can significantly impair visual acuity if not adequately treated. Fluocinolone-acetonide sustained-release-0.2 µg/day intravitreal (FAc) implants are indicated for prevention of relapse in recurrent NIU-PS. The aim here was to provide treating clinicians with some consensus-based-recommendations for the clinical management of patients with NIU-PS with 0.2 µg/day FAc implants.

**Methods:**

A European-clinical-expert-group agreed to develop a consensus report on different issues related to the use of FAc implants in patients with NIU-PS.

**Results:**

The Clinical-expert-panel provided specific recommendations focusing on clinical presentation (unilateral/bilateral) of the NIU-PS; systemic involvement of NIU-PS and the lens status. Treatment algorithms were developed; one that refers to the management of patients with NIU-PS in clinical practice and another that establishes the best clinical scenarios for the use of FAc implants, both as monotherapy and as adjuvant therapy. Additionally, the Clinical-expert-panel has provided recommendations about the use of the FAc implants in a clinical-setting. The Clinical-expert-panel also considered the safety profile of FAc implants and their possible implications in the daily practice.

**Conclusions:**

As more clinical experience has been gained using FAc implants, it was necessary to update the clinical recommendations that guide patient management in the clinic. The current consensus document addresses relevant issues related to the use of FAc implants on different types of patients with various etiologies of NIU-PS, and was conducted to standardize approaches to help specialists obtain better clinical outcomes.

**Supplementary Information:**

The online version contains supplementary material available at 10.1186/s12348-024-00402-4.

## Background

Non-infectious uveitis affecting the posterior segment of the eye (NIU-PS) is an inflammatory disease, which may result in severe visual impairment and tissue damage when it is not adequately treated and controlled [1]. Additionally, the prevalence of bilateral involvement is high, and it is estimated that it accounts for approximately 10%-15% of cases of blindness in developed countries [2]. Unlike other retinal diseases, uveitis is the fifth most common cause of vision loss in high-income countries, accounting for 5% to 20% of legal blindness [3, 4], with the highest incidence of disease in the working-age population [5] with significant social and economic impact [6, 7].

Many etiologies have been associated with NIU-PS, including Birdshot retinochoroiditis, Behçet’s disease, sarcoidosis, and intermediate uveitis [2, 8, 9].

Current treatment strategies of NIU-PS attempt to control active inflammation and to prevent recurrences. This is done to avoid the potential reduction of visual function and is initially achieved using systemic corticosteroids and then secondary using immunosuppressive agents as they are steroid sparing agents. Since both local and/or systemic treatments may be associated with significant side effects, new strategies for delivering the drug to the site of inflammation, the vitreous cavity, have been developed [10, 11].

Fluocinolone acetonide sustained-release 0.2 µg/day intravitreal (FAc) implants (ILUVIEN®; Alimera Sciences, Dublin, Ireland and YUTIQ®; Alimera Sciences Inc., Alpharetta, Georgia, USA) are indicated for the prevention of relapse in recurrent NIU-PS [12] and for the for the treatment of chronic non-infectious uveitis affecting the posterior segment of the eye [13], respectively.

The results of a pilot study, conducted on eleven eyes from 11 patients with a history of recurrent NIU-PS, showed FAc implants improved the best-corrected visual acuity (BCVA) from 0.56 ± 0.43 logMAR at baseline to 0.17 ± 14 logMAR at month-24 (*p* = 0.0016). None of the studied eyes experienced a recurrence during the follow-up period [14].

Additionally, the 36-month effectiveness and safety of FAc implants were evaluated in a phase 3, prospective, double-masked, and multicenter study [15]. Patients with NIU-PS were randomly assigned to receive treatment with the 0.2 μg/day FAc implant or Sham treatment (sham injection plus standard of care) treatment. BCVA significantly improved by + 9.1 letters in the FAc implant group compared with + 2.5 letters for the sham-treated group (*p* = 0.020). Over 36 months the cumulative uveitis recurrence rate was significantly lower in the FAc- implant group (65.5%) compared with 97.6% in the sham-treated group (*p* < 0.001) [15]. At month 36, the time to first recurrence in FAc-treated eyes was significantly longer compared with sham-treated eyes (median 657.0 days and 70.5 days, respectively; *P* < 0.001). Moreover, the number of recurrences per eye occurring over 36 months was also significantly lower in the FAc-treated group compared with the sham-treated group (mean 1.7 vs. 5.3 respectively, *P* < 0.001) [15] Intraocular pressure was well controlled in both study groups and approximately half as many eyes in the FAc-treated group when compared with the sham-treated group underwent IOP-lowering surgery (5.7% vs. 11.9%) [15].

Despite the good clinical outcomes reported in these trials, they did not provide any information about the etiology of the NIU-PS [14, 15]. Since FAc implants have become available in the USA and some European countries, more clinical experience has been gained and more studies evaluating FAc implants in different clinical scenarios have been published [15–28] showing that the FAc implants are effective for preventing recurrence of ocular inflammation in patients with NIU-PS [14–29].

Despite this evidence, there are still certain doubts regarding the clinical management of NIU-PS in daily clinical practice using the 0.2ug/day fluocinolone acetonide intravitreal implants.

The aim of this review was to generate consensus-based recommendations from a group of uveitis experts with substantial experience of treating NIU-PS with 0.2ug/day FAc implants in their clinical practice.

## Methods

A European Clinical expert group of 10 Uveitis/retinal specialists from France; Germany; Italy; Spain; and United Kingdom, with significant experience in using the FAc implant in patients with NIU-PS, was formed to collaborate to develop a consensus report on different issues related to the most valuable applications on the use of the FAc implants (ILUVIEN®; Alimera Sciences Ltd., Hampshire, UK and YUTIQ®; Alimera Sciences Inc., Alpharetta, Georgia, USA) in patients with different clinical presentations and etiologies of NIU-PS in daily practice.

This project was carried out in six phases: (1) Initial phase, where the expert panel members reviewed the currently available scientific evidence. (2) The panel selected and agreed different topics relating to the use of FAc implants in patients with NIU-PS and developed a questionnaire; (3) the panel of experts answered the questionnaire; (4) the experts reviewed and discussed the results of the survey in a virtual meeting held on April 2023; (5) The panel performed a second round of the survey; and (6) The panel reviewed, analyzed, and validated the data from the second-round survey in a virtual meeting held on May 2023.

The degree of consensus was determined at the end of the process (Table S1).

### Scientific evidence

A literature search of Pubmed/MEDLINE conducted by using a combination of keywords related to uveitis (non-infectious/non-infectious uveitis; inflammation; Behçet disease; sarcoidosis uveitis; birdshot retinochoroiditis; post-surgical macular edema; prevalence; incidence; corticosteroids). The search period ranged from January 2000 to June 2023. References cited in the individual papers were also reviewed to identify any relevant reports. In addition, relevant national and international guidelines were reviewed.

### Questionnaire development

After reviewing the literature, the Clinical expert panel identified and discussed those aspects that may generate expert discussion and controversy, and decided which were to be included in the questionnaire.

The questionnaire included 21 items involving the use of the FAc implants and the management of patients with NIU-PS (see Annex I).

## Results

### Survey

The survey aimed at answering the diverse queries that arise about the clinical management of patients with NIU-PS and the use of the 0.2 µg/day FAc implants in these patients in clinical practice.

Table 1 shows the expert panel responses to the different survey questions after completing the first and second survey.

**Table 1:** Overview of the expert panel responses to the different survey questions after the first and second rounds

	**Answer: Yes, %**			**Comments**	
	**First round**	**Second round**	**∆**	**DoA**	
Item 1	100	100	0	Strong	Depends on the patient’s lens status and previous treatments In monotherapy or in combination with systemic treatment depending on each specific case
Item 2	100	100	0	Strong	
Item 3	80	100	+ 20	Strong	
Item 4	90	100	+ 10	Strong	In monotherapy or in combination with systemic treatment depending on the presence of deep choroidal inflammation
Item 5	90	80	+ 10	Agreement	Combination with systemic therapy to control the intraocular inflammation
Item 6	60	80	+ 20	Agreement	The age of the patient, uveitis severity and the need for combination therapy need to be considered FAc 190 µg would be considered in the presence of presbyopia, cataract, or planned cataract surgery after the implant
Item 7	100	100	0	Strong	
Item 8	10	0	-10	Strong	Risk of anterior chamber migration Periocular triamcinolone may present a better option in these eyes FAc 190 µg would be considered if sutured to the sclera
Item 9	60	70	+ 10	MA	Except in primary choroiditis or NIU etiologies of transient nature (e.g., MEWDS)
Item 10	70	90	+ 20	Agreement	Except in primary choroiditis Would not be considered first-line in acute phase of inflammation Use to maintain quiescence and control the macular edema
Item 11	10	20	+ 10	Agreement	No, as it is also effective at controlling inflammation in the vitreous and in retinal vasculitis which may not always be associated with uveitic macular edema
Item 12					
Inject DEX-i	90	90	0	Agreement	Dexamethasone implant is considered first-line to control the active ocular inflammation If recurrent inflammation 3—4 months with 1 or 2 successive dexamethasone implants, use of fluocinolone acetonide 190 µg intravitreal implant to maintain/achieve quiescence
Inject PTA	20	20	0	Agreement	
Inject ITA	20	0	-20	Strong	
Inject FAc 190 µg	80	80	0	Agreement	
Item 13					Use of FAc 190 µg to maintain/achieve quiescence
A	0	0	0	Strong	
B	0	0	0	Strong	
C	40	40	0	MA	
D	100	100	0	Strong	
E	0	0	0	Strong	
Item 14					There is lack of evidence on the effectiveness of intravitreal steroids in choroidal neovascularization The age of the patient and systemic treatment needs to be considered and reviewed It is suggested that in the presence of choroidal neovascularization, treatment could implicate combination of anti-VEGFs and intravitreal steroids, together with a review of the systemic therapy
A	0	0	0	Strong	
B	0	0	0	Strong	
C	20	40	+ 20	MA	
D	10	50	+ 40	NA	
E	50	50	0	NA	
Item 15					Local intraocular steroids would only be considered as an adjunctive therapy in controlling PSME, optic disc swelling, vitritis and retinal vasculitis
A	0	0	0	Strong	
B	0	0	0	Strong	
C	30	20	-10	Consensus	
D	10	20	+ 10	Consensus	
E	80	60	-20	MA	
Item 16					Dexamethasone implant
A	20	0	-20	Strong	
B	0	0	0	Strong	
C	100	90	-10	Consensus	
D	10	10	0	Consensus	
Item 17					In remission on treatment In remission off treatment: according to the SUN working group—Inactive disease for ≥ 3 months after discontinuing all treatments for eye disease
Inactivity of recent onset	30	30	0	MA	
In remission ON treatment	90	80	-10	Consensus	
In remission OFF treatment	60	60	0	MA	
Item 18					After recurrence of inflammation and/or macular edema to local therapy After recurrence of inflammation and/or macular edema to systemic therapy The use of fluocinolone acetonide 190 µg intravitreal implant could be considered before recurrence of inflammation and/or macular edema to local therapy if there is previous knowledge of the dexamethasone implant response
F	70	70	0	MA	
G	40	40	0	MA	
H	60	70	+ 10	MA	
I	30	30	0	MA	
Item 19					
BSR	100	100	0	Strong	
APMPPE	30	30	0	MA	
MEWDS	10	10	0	Agreement	
MCP	100	100	0	Strong	
SFUS	70	70	0	MA	
ARPE	30	30	0	MA	
PIC	80	80	0	Agreement	
AZOOR	70	70	0	MA	
SC	90	90	0	Agreement	
Behçet disease	80	80	0	Agreement	
SO	80	80	0	Agreement	
Sarcoidosis	100	100	0	Strong	
Intermediate uveitis	90	90	0	Agreement	
VKH disease	70	70	0	MA	
TINU	50	50	0	NA	
PSCME	90	90	0	Agreement	
Item 20					
BSR	20	20	0	Agreement	
APMPPE	20	20	0	Agreement	
MEWDS	0	0	0	Strong	
MCP	50	50	0	NA	
SFUS	40	40	0	MA	
ARPE	20	10	0	Agreement	
PIC	40	40	0	MA	
AZOOR	30	30	0	MA	
SC	30	30	0	MA	
Behçet disease	20	20	0	Agreement	
SO	10	10	0	Agreement	
Sarcoidosis	70	70	0	MA	
Intermediate uveitis	70	70	0	MA	
VKH disease	20	20	0	Agreement	
TINU	50	50	0	NA	
PSCME	90	90	0	Agreement	
Item 21					Depends on clinical findings and recurrence history of the patient
< 3 years	70	80	+ 10	Agreement	
≥ 3 years	90	60	-30	MA	
Item 22					Recurrence of ocular inflammation Recurrence of macular edema
ROI	100	100	0	Strong	
RME	100	100	0	Strong	
QoE	10	20	+ 10	Agreement	
J	0	0	0	Strong	

*FAc* Fluocinolone acetonide 0.2 µg/day intravitreal implant, *NIU* Non-infectious uveitis, *DoA* Degree of agreement, *MA* Majority agreement, *NA* Not agreement, *MEWDS* Multiple Evanescent White Dot Syndrome, *DEX-I* Intravitreal dexamethasone implant, *PTA* Periocular triamcinolone acetonide, *ITA* Intravitreal triamcinolone acetonide, *PSCME* Post-surgical cystoid macular edema, *BSR* Birdshot retinochoroiditis, *MCP* Multifocal choroiditis and panuveitis, *APMPPE* Acute posterior multifocal placoid pigment epitheliopathy, *SFUS* Subretinal fibrosis and uveitis syndrome, *ARPE* Acute retinal pigment epitheliitis, *PIC* Punctate inner choroiditis, *AZOOR* Acute zonal occult outer retinopathy, *SC* Serpiginous choroiditis, *SO* Sympathetic ophthalmia, *VKH* Vögt-Koyanagi-Harada disease, *TINU* Tubulointerstitial nephritis and uveitis, *ROI* Recurrence of ocular inflammation, *RME* Recurrence of macular edema, *QoE* Quiescence of the eye

A: PTA would be chosen as preferable treatment

B: ITA would be chosen as preferable treatment

C: DEX-i would be chosen as preferable treatment

D: FAc would be chosen as preferable treatment

E: I would not expect local corticosteroids to be effective

F: After recurrence of inflammation and/or macular edema to a local steroid

G. Before recurrence of inflammation and/or macular edema to a local steroid

H: After recurrence of inflammation and/or macular edema to systemic therapy

I: Before recurrence of inflammation and/or macular edema to systemic therapy

J: I would not consider re-injection

The different survey questions could be framed in the following subjects:

- Unilateral/Bilateral NIU-PS

According to the panel, 0.2 µg/day FAc implants can be used in cases of unilateral or asymmetric inflammation without associated systemic involvement, contraindications to systemic treatments, refractory damage and/or macular oedema. In cases of bilateral symmetric involvement and/or associated systemic involvement, the first course of treatment is general systemic treatment followed by a reduction, more or less associated with sparing treatment in cases of corticosteroid dependence. The use of 0.2 µg/day FAc implants in monotherapy or in combination with systemic treatment will depend on the patient’s lens status and previous treatments.

- NIU-PS in relation to systemic involvement

0.2 µg/day FAc implants might be used in eyes with NIU-PS without systemic involvement, either in monotherapy or in combination with systemic treatment, depending on the presence of deep choroidal inflammation (100% agreement). On the other hand, 0.2 µg/day FAc implants could be chosen in uveitis in association with systemic involvement in combination with systemic therapy to control the ophthalmic inflammation. (e.g., in Behcet disease patients) (90% agreement).

- Lens status

The panel considered that 0.2 µg/day FAc implants can be used in pseudophakic eyes with NIU-PS (100% agreement), but not in aphakic eyes with NIU-PS (100% agreement). According to the panel, 0.2 µg/day FAc implants might be used in phakic eyes with NIU-PS (80% agreement), although patient age (young patients), uveitis severity (greater inflammatory activity), and the need for combination therapy need to be considered. In addition, the use of 0.2 µg/day FAc implants would be considered in patients with presbyopia, cataract, or planned cataract surgery after the implant.

### Treatment algorithms and recommendations

The panel has developed two fundamental treatment algorithms, based both on the currently available scientific evidence and on the experience of its members. One that refers to the practical management of patients with NIU-PS in clinic and another that establishes the best clinical scenarios for the use of 0.2 µg/day FAc implants, both as monotherapy and as combination therapy.

Additionally, the panel has developed a table that includes the main uveitic etilogies for use of the 0.2 µg/day FAc implants in a clinical setting.

**Fig. 1:**
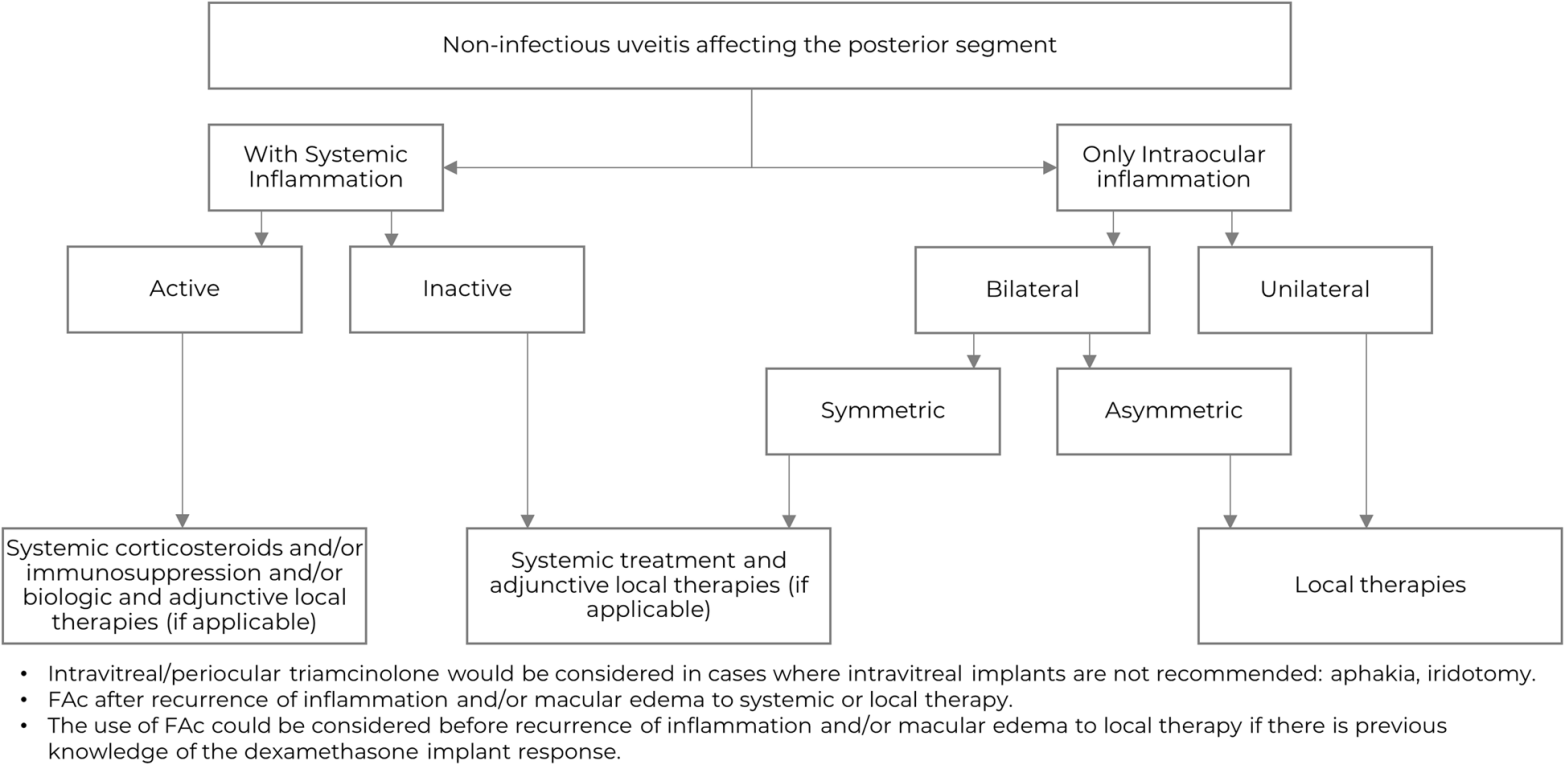
Treatment algorithm of eyes with non-infectious uveitis affecting the posterior segment. FAc: Fluocinolone acetonide sustained-release 0.2 µg/day intravitreal implant

Figure 1 shows the treatment algorithm of eyes with NIU-PS. This algorithm considered various aspects relating to the disease, such as the presence of intraocular inflammation with or without systemic inflammation, the presence or absence of active systemic inflammation, or its bilaterality, amongst others.

According to the panel's recommendations:

- Intravitreal/periocular triamcinolone (which is off-label in many countries) would be considered in cases where intravitreal implants are not recommended: e.g., aphakia, large iridectomy.
- To use 0.2 µg/day FAc implants after recurrence of inflammation and/or macular edema as adjunctive to systemic or topical therapy.
- The use of 0.2 µg/day FAc implants could be considered before recurrence of inflammation and/or macular edema to local therapy if there is previous knowledge about the patient’s response to a prior dexamethasone implant (DEX-i).

According to the panel, FAc implant would be considered for use in patients in:

- Unilateral NIU-PS.
- Bilateral asymmetrical NIU-PS.
- Bilateral symmetrical NIU-PS.
- NIU-PS with no systemic involvement.
- NIU-PS with systemic involvement as an adjunctive therapy.
- NIU-PS with deep choroidal involvement as an adjunctive therapy in cases of retinal or superficial choroidal inflammation.
- Pseudophakia or pre-existing cataract.
- NIU-PS with macular edema.
- Systemic treatment burdensome or contraindicated.

**Fig. 2:**
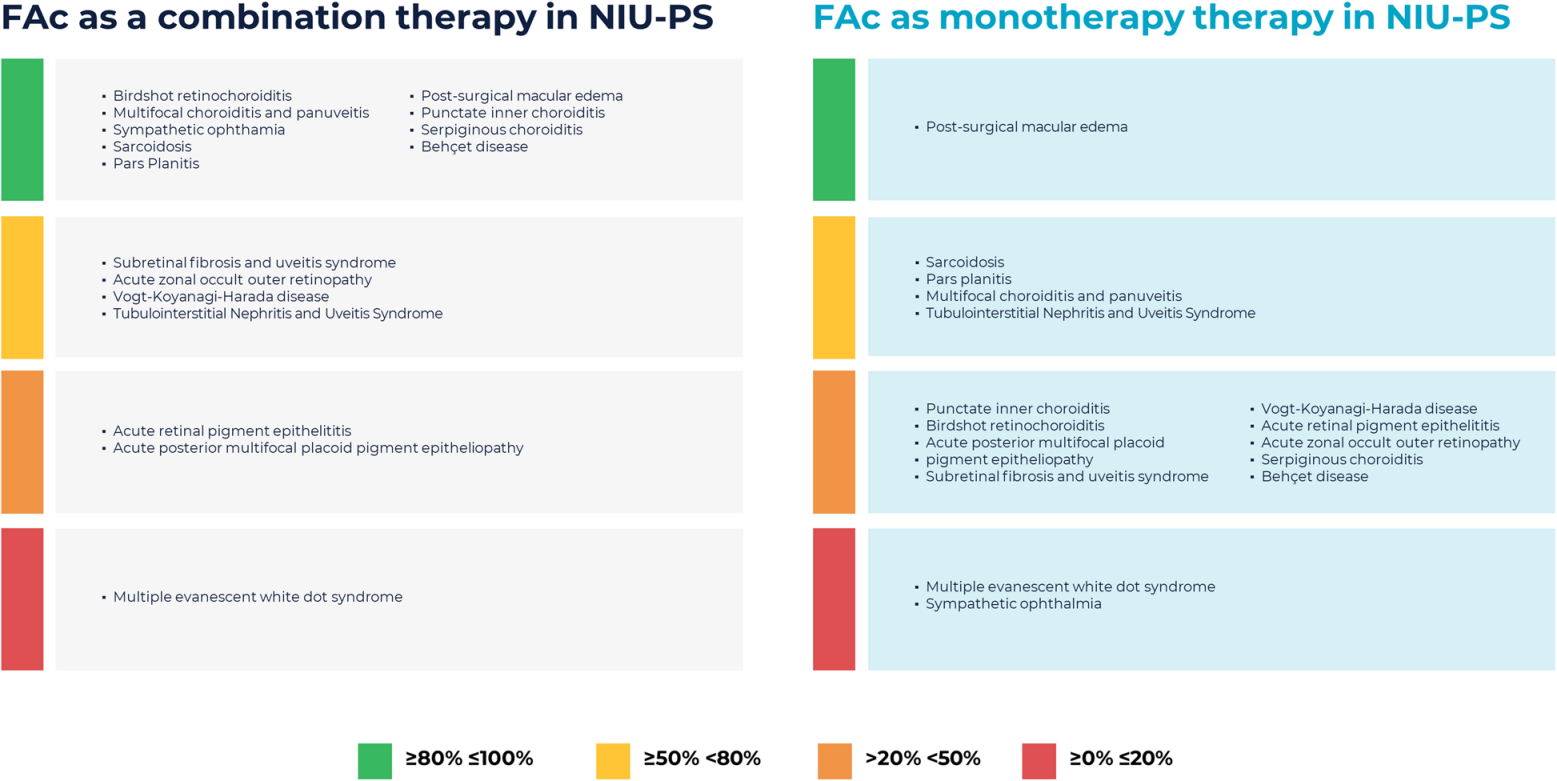
Degree of agreement of the members of the panel about the use of the fluocinolone acetonide 0.2 µg/day intravitreal (FAc) implant among the different etiologies associated with the onset of non-infectious uveitis affecting the posterior segment (NIU-PS) of the eye. *Macular edema includes Cystoid macular edema and diffuse macular edema (this is not frequent). FAc: Fluocinolone acetonide 0.2 µg/day intravitreal implant; NIU-PS: Non-infectious uveitis affecting the posterior segment

Figure 2 shows the degree of agreement of the members of the panel about the use of the FAc implant amongst the different NIU-PS etiologies.

The Table 2 summarizes the role of FAc implant in different NIU-PS etiologies associated with the onset of NIU-PS.

**Table 2:** Role of the Fluocinolone acetonide 0.2 µg/day intravitreal (FAc) implant in the different etiologies associated with the onset of non-infectious uveitis affecting the posterior segment (NIU-PS) of the eye

Etiology	Comments
Birdshot retinochoroiditis	FAc implant has an adjunctive role in controlling PSME, optic disc swelling, vitritis, and retinal vasculitis
Multifocal choroiditis and panuveitis	
Intermediate uveitis	
Sympathetic ophthalmia	FAc implant has an adjunctive role in controlling PSME, optic disc swelling, vitritis and retinal vasculitis FAc implant has an adjunctive role in controlling subretinal inflammation
Sarcoidosis	
Post-surgical ME	FAc implant has an adjunctive role in treating and preventing recurrence of recurrent PSME after 1–2 dexamethasone implant
Punctate inner choroiditis	FAc implant has an adjunctive role in unilateral cases FAc implant could be considered in cases where systemic therapy is not recommended or contraindicated
Serpiginous choroiditis	FAc implant has an adjunctive role in case of recurrent inflammation
Behçet disease	
Subretinal fibrosis and uveitis syndrome	FAc implant has an adjunctive role in case of recurrent inflammation and subretinal component as well
Acute zonal occult outer retinopathy	FAc implant has an adjunctive role in case of recurrent inflammation
Vögt-Koyanagi-Harada disease	FAc implant has an adjunctive role for the retinal/subretinal component (vasculitis, pseudo-dallen-fuchs), optic disc swelling, relapsing iridocyclitis or eventual vitreous inflammation
TINU	FAc implant has an adjunctive role for the retinal/subretinal component if present, optic disc swelling, relapsing iridocyclitis or eventual vitreous inflammation (haze) as well
Acute retinal pigment epitheliitis	FAc implant has an adjunctive role in case of recurrent inflammation or possible choroidal neovascularization
Acute posterior multifocal placoid pigment epitheliopathy	FAc implant could be considered in cases where systemic therapy is not recommended or contraindicated
Multiple evanescent white dot syndrome	FAc implant has not an adjunctive role as this disease is self-limiting

*FAc* Fluocinolone acetonide 0.2 µg/day intravitreal, *PSME* Post-surgical macular edema, *TINU* Tubulointerstitial nephritis and uveitis

As a combination therapy, the FAc implant would be a valuable treatment option in the following etiologies: Birdshot retinochoroiditis, multifocal choroiditis and panuveitis, sarcoidosis, intermediate uveitis, post-surgical macular edema (PSME), serpiginous choroiditis, or Behçet’s disease, among others (degree of agreement ≥ 80% each, respectively). However, its use in eyes with multiple evanescent white dot syndrome was not considered a good option (degree of agreement ≥ 0% ≤ 20%) (Fig. 2 and Table 1).

As a monotherapy, the FAc implant could be considered in post-surgical macular edema (degree of agreement ≥ 80%), as well as in sarcoidosis, intermediate uveitis, and multifocal choroiditis and panuveitis (degree of agreement ≥ 50% < 80% each) (Fig. 2 and Table 1).

## Discussion

Treatment options for NIU-PS include corticosteroids (systemic, topical, periocular, intravitreal), antimetabolites, calcineurin inhibitors, alkylating agents, and biological agents [11, 30–35]. Among them, systemic corticosteroids are considered as the first line treatment for NIU-PS, due mainly to their efficacy and rapid control of inflammation [11, 33, 34]. Corticosteroids can be administered topically, periocularly, intraocularly, or systemically, depending on the disease severity and the type of ocular involvement [11, 33, 34]. However, the prolonged use of systemic therapies, including corticosteroids, classic immunomodulators, and biologics, have been associated with both systemic (e.g., diabetes, Cushing's syndrome, major psychiatric disorders, or gastroduodenal ulcer) and ophthalmological (cataract, raised intraocular pressure) [11, 36]; while short-acting localized corticosteroids are problematic as they are short-acting and do not control the underlying inflammation or flare-ups that occur with NIU-PS, with oscillating periods of functional and structural amelioration and worsening, which means a greater probability of cumulative and permanent visual damage [37].

Since NIU-PS is a chronic disease, long-lasting therapeutic options, which minimize the incidence of recurrences and systemic/local side effects, are highly desirable [24].

Recent technology has permitted the development of long-lasting low-dose sustained release intravitreal corticosteroid implants which have changed the treatment paradigm of NIU-PS [10]. Compared to systemic, periocular, and other intravitreal corticosteroids (i.e., triamcinolone acetonide and dexamethasone implant), 0.2 µg/day FAc implants offer the advantage of a gradual and sustained localized release of the corticosteroid to the posterior segment of the eye, resulting in reduced rates of relapses and fewer injections, and significantly reducing the systemic immunomodulation load requirements [10, 11].

In a systematic review published in 2021, the main conclusion drawn from the survey, regarding effectiveness, was that implants reduced the incidence of recurrences and the need for adjuvant therapies [38]. This suggests clearly the systemic immunomodulation sparing effect that slow-release local therapies have. Regarding safety, adverse events were as expected and they were safely managed within the studies [38].

Up to now, there are four sustained release intravitreal corticosteroid implants, one of dexamethasone 700 µg (Ozurdex®, AbbVie Company, Dublin, Ireland); and three of fluocinolone acetonide with different doses, namely 0.59 mg (Retisert®, Bausch and Lomb, Inc. USA), 180 µg (Yutiq®; Alimera Sciences Inc., Alpharetta, Georgia, USA), and 190 µg (ILUVIEN®; Alimera Sciences, Dublin, Ireland). ILUVIEN® is currently available in EU and the United Kingdom.

The intravitreal 0.59-mg fluocinolone acetonide implant (Retisert®, Bausch and Lomb, Inc. USA) was the first US FDA-approved implant for treatment of NIU-PS, but it requires surgical implantation and has been associated with several complications (hypotony, resistant intraocular pressure elevation, endophthalmitis). In addition, the use of Retisert® (Bausch and Lomb, Inc. USA) is only available in the USA, since it has not been approved in Europe.

Both 190 µg and 180 µg fluocinolone acetonide implants are injected intravitreally, by a preloaded applicator with a 25-gauge needle through the pars plana. Both implants were designed to release fluocinolone acetonide at a rate of 0.2 μg/day over a 3-year period, progressively decreasing to 0.1 μg/day [12, 39–41].

An overview of published evidence for the 0.2 µg/day FAc implant is summarized in Table 3.

**Table 3:** Summary of published studies for injectable 0.2 µg/day fluocinolone acetonide implants in non-infectious uveitis

Study	190 µg Fluocinolone Acetonide Intravitreal implant (Alimera Sciences, Hampshire, UK)			
	**N**	**Follow-up**	**Diagnosis**^**a**^	**Main Outcomes**
Jaffe et al. [14]	11	24 M	Idiopathic; MS; Sarcoidosis; PsA; JIA	1. Mean study eye VA improved from + 0.56 to + 0.25 and + 0.17 logMAR at 12 and 24 months after implantation, respectively (*P* = 0.041 and *P* = 0.016) 2. The average number of inflammation recurrences in the 12 months before implantation was 1.54 episodes per eye. None of the study eyes experienced a recurrence during the follow-up period 3. Five of 11 eyes received an average of 1.6 posterior sub-Tenon triamcinolone acetonide injections in the 12 months preceding implantation. None required a PSTA injection after FAc implantation
Jaffe et al. [15]	87	36 M	Idiopathic; MS; Sarcoidosis; PsA; JIA	1. BCVA improved + 9.1 letters in the FAc group 2. Only 13% of the eyes had investigator-determined macular edema at month 36 in the FAc group 3. Recurrence rate significantly lower in the FAc group (5.7%) than in the sham group (28.6%), *p* < 0.001 4. Fewer eyes required adjunctive treatments in the FAc group
Meira et al. [16]	1	36 M	SO	1. BCVA improved from 20/200 to 20/50 2. CFT decreased from 490 μm to 153 μm 3. IOP maintained normal, without the need for medication
Weber et al. [17]	11	19 M	Idiopathic; RA; MS; MCP; AZOOR	1. 82% of eyes improved BCVA (between + 1 and + 8 lines) 2. CFT decreased to 168 μm 3. 82% presented with inactive inflammation during the follow-up period 4. The mean IOP increase was 2.1 ± 4.7 mmHg 5. Two phakic patients developed a cataract and underwent cataract surgery
Pockar et al. [18]	11	12 M	Idiopathic; Sarcoidosis; SLE	1. BCVA was stable 2. CRT decreased from 435 μm to 296 μm 3. IOP > 21 mmHg was observed in three eyes, and > 30 mmHg in one eye, managed with topical therapy 4. Two eyes received adjunctive treatment for worsening CRT
McGregor et al. [19]	2	36 M	Idiopathic	1. There was a rapid clinical response with resolution of hypopyon OS. Remission was mostly maintained for 3 years 2. During all treatments, there was only 1 IOP rise event (24 mm Hg) and that preceded treatment with intravitreal steroid and was associated with a uveitis flare in the patient's right eye 3. Central retinal thickness demonstrated resolution of CME
Ansari et al. [20]	2	12 M	JIA	1. The LE showed an improvement in VA to 0.42 from 0.98 logMAR 2. CRT decreased from 471 μm to 272 μm (LE) 3. The RE showed an improvement in VA to 0.10 from 0.56 LogMAR 4. CRT decreased from 590 μm to 263 μm (RE—6 months FU)
Hikal et al. [21]	34	18 M	Idiopathic; RA; MS; Sarcoidosis; BRC; MCP; AZOOR; IU	1. Macular edema was completely resolved in70.6% cases treated 2. In 58.5%, VA improved (from + 1 to + 5 lines) and remained stable in 26.5% 3. Five of the treated eyes had a relapse after 23.2 ± 14 months 4. Three FAc reinjections were performed and a drying of the macula was observed 5. Three of these eyes had a cataract prior to therapy and the other developed a cataract 2.5 years after the FAc implant was administered
Moreno-Castro et al. [22]	2	24 M	IRVAN	1. A decrease in macular thickening was observed in both eyes 2. BCVA was 20/30 (RE) and 20/60 (LE) during follow-up 3. IOP was 16 mmHg in both eyes with the need for medication
Ajamil-Rodanes et al. [23]	15	31 M	BRC	1. Between month 6 and 12, FA showed that 73.4% of eyes had no leakage, this increased to 84.6% by month 24 2. Three eyes had CMO at baseline. 6 months after FAc implant, all eyes achieved complete CMO resolution 3. One year after insertion of the implant, the characteristic hypofluorescent lesions on ICGA were unchanged in all cases 4. Retinal function improved and macular function improved or was stable in the majority following treatment
Pavesio & Heinz [24]	59	36 M	Idiopathic; MS; Sarcoidosis; PsA; JIA	1. BCVA improved + 9.6 letters in the FAc group 2. Mean number of recurrences was 1.9 in the FAc group 3. 18 eyes in the FAc group required cataract surgery
Studsgaard et al. [25]	20	24 M	Idiopathic; RA; Sarcoidosis; GPA; JIA; BRC; MCP; SO	1. BCVA improved at 24 months and CRT decreased as well 2. No patients started new systemic treatment 3. Eight eyes were treated with topical IOP-lowering medication at the time of implantation, of these two later underwent trabeculectomy. There were no complications associated with previous glaucoma surgery
Battista et al. [26]	10	12 M	Idiopathic; Sarcoidosis; SC; BD	1. The area under the curve for BCVA significantly improved from month 6 (*p* = 0.03) 2. The CMT improved from month 1 and was persistently lower than baseline until month 12 (*p* < 0.001) 3. No adverse events were recorded over 1 year
Kessler et al. [27]	29	42 M	Idiopathic; MS; Sarcoidosis; BRC; MCP; AZOOR; VKH; IU	1. The number of corticosteroids (CS) required prior to FAc injection predicted the need for additional CS after therapy with the implant 2. In contrast, a higher decrease in choroidal vascularity index (CVI) at 6 months after FAc therapy commenced was negatively correlated to the number of additional CS needed after the implant was given. These parameters may anticipate the need for adjunctive CS
Kriegel et al. [28]	23	1.7 M	Sarcoidosis; BRC	1. CST (Dex: *p* < 0.0001; FAc: *p* = 0.0008) and BCVA (Dex: *p* = 0.0009; FAc: *p* = 0.0005) improved significantly with both implants 2. Significantly better effects were noted with Dex for absolute and relative CST reduction (*p* = 0.0089 and *p* = 0.0051, respectively). Final BCVA did not differ between groups (*p* = 0.1893) 3. Dex significantly increased IOP, whereas FAc did not 4. One eye was actively inflamed after Dex and FAc injection at follow-up (inflamed eyes before injection: [Dex: 2; FA: 6])
Buhl et al. [29]	76	12 M	Sarcoidosis; BRC	1. BCVA remained stable 2. CRT reduction (362.7 vs 309.1 μm; *p* = 0.04) 3. Reduced intraocular inflammation (0.82 vs 0.3; *p* = 0.007) 4. IOP increase (13.68 vs 15.6; *p* = 0.0507) 5. Cataract development (20% of phakic eyes)
Reddy et al. [42]	1	8 M	Idiopathic	1. BCVA improved from 20/200 to 20/20 2. CMT decreased from 707 μm to 364 μm 3. IOP maintained normal
Moll-Udina et al. [43]	26	12 M	Idiopathic, sarcoidosis, BRC, post-surgical uveitis, TINU, IRVAN, Blau syndrome-associated uveitis	1. BCVA was significantly improved at all the time point measured (*p* < 0.01 each) 2. CMT was significantly reduced at all the time point measured (*p* < 0.01 each) 3. Systemic corticosteroid dose pre-FAc implant, higher immunomodulatory therapy load at baseline, and thicker retinal nerve fiber layer at baseline were predictors of FAc implant effectiveness at month-12 4. IOP remained stable throughout the study
Buhl et al. [44]	50	36 M	Idiopathic	1. BCVA and CRT remained stable until month 36 after FAc implant injection 2. Recurrence rate was 34% (17/50) eyes, of which, 14 eyes received high-dose corticosteroids before FAc implant injection 3. IOP remained unchanged 4. Cataract surgery was performed in 13 of the 14 phakic eyes
Marques et al. [45]	5	36 M	PSCME^b^	1. BCVA improved from 0.3–0.3 LogMAR to 0.4 – 0.3 LogMAR 2. CMT decreased from 492 – 38.0 µm to 369.0–324.0 µm 3. IOP increase from 16.0–0 mmHg to 17.0–3.0 mmHg. Four of five eyes had increased IOP and were managed with intraocular pressure-lowering eye drops
Chronopoulos et al. [46]	16	24 M	PSCME	1. At month 24, BRVA improved in 5 eyes, remained stabilized in 5 eyes, and decreased in 1 eye 2. Mean CRT decreased from 524 ± 132 μm at baseline to 313 ± 83 μm (*p* = 0.0001) at month 24 3. Increased IOP (≥ 21 mmHg) was observed only in 4 eyes, all successfully managed with ocular hypotensive medication
Lima-Fontes et al. [47]	9	44 M	Recurrent PSCME	1. Mean BCVA improvement from baseline was 17.2 ± 10.0 letters 2. Mean CMT reduction from baseline was 208.2 ± 180.4 µm 3. IOP-lowering regimen was increased in one eye and two additional eyes started hypotensive drops
Kessler L et al. [48]	23	24 M	Idiopathic; MS; Sarcoidosis; BRC; MCP; AZOOR; VKH; IU	1. BCVA and CMT significantly improved after FAc implantation (*P* < 0.05) 2. AUCBCVA and AUCCMT were 0.41 – 0.33 LogMAR of resolution/6 months and 320.15 – 321.64 µm/6 months, respectively 3. Better baseline BCVA (coefficient [coef.] = 0.83, *P* < 0.001) and macular thickness reduction after FAc administration (coef = -0.0001, *P* < 0.05) were associated with better BCVA after FAc treatment 4. In contrast, baseline OCT biomarkers such as ellipsoid zone reflectivity and choroidal vascularity index, sex, or disease duration before FAc injection showed no correlation with AUCBCVA and AUCCMT (*P* > 0.05) 5. The younger the patient at the time of FAc injection, the greater the reduction in CMT (coef. = 1.76, *P* < 0.05)
Ong S et al. [49]	1	11 (RE) – 13 (LE) M	PSCME after vitrectomy	1. VA improved from 20/126 to 20/50 in the RE and 20/80 to 20/40 in the LE 2. Central subfield thickness decreased from 592 µm to 288 µm in the RE and 565 µm to 287 µm in the LE, without IOP elevation
Alfaqawi F, et al. [50]	1	20 M	PSCME after Retinal detachment repair	1. VA improved to 6/18 2. CME was resolved 3. IOP increased to 27 mmHg and regressed with IOP medication
Herold TR, et al. [51]	2	1 M	PSCME after disrupted anterior–posterior segment border	1. Both patients showed first morphological improvement in terms of reduction of CRT in the first 4 weeks after the procedure 2. BCVA increased in one patient by one line and remained stable in the other patient in the first 4 weeks of the follow-up period
Miguel-Escuder L, et al. [52]	4	11-22 M	PSCME after retinal detachment repair (*n* = 2) After vitrectomy (*n* = 1) After cataract surgery (*n* = 1)	Case 1. BCVA improved from 20/40 to 20/20; CRT decreased by –246 μm; maximum IOP was 18 mmHg Case 2. BCVA improved from 20/200 to 20/40; CRT decreased by –151 μm; maximum IOP was 26 mmHg and regressed with IOP medication Case 3. BCVA improved from 20/200 to 20/63; CRT decreased by –364 μm; maximum IOP was 25 mmHg and regressed with IOP medication Case 4. BCVA improved from 20/50 to 20/32; CRT decreased by – 62 μm; maximum IOP was 15 mmHg
Herold TR, et al. [53]	10	24	PSCME after cataract surgery	1. A significant improvement to 0.57 ± 0.38 log MAR (Snellen 20/80) (range 0–1.30) was observed (*P* = 0.003) at 1 month. Further improvement to 0.45 ± 036 logMAR (Snellen 20/60) was observed until month 18 (*P* = 0.081) 2. Mean central retinal thickness decreased by 22% from 601.6 ± 235.5 mm to 449.1 ± 128.9 mm at 1 month 3. In one patient, the implant has to be removed at Month 7 because of elevated intraocular pressure and one patient after globe rupture had a retinal redetachment at Month 4
	**180 µg Fluocinolone Acetonide Intravitreal implant (Alimera Sciences Alpharetta Georgia, USA)**			
**Study**	**N**	**Follow-up**	**Diagnosis**^**a**^	**Main Outcomes**
Kiernan DF, et al. [54]	2	6-15 M	PSCME after cataract surgery	Case 1. BCVA improved from 20/70 to 20/25; CST decreased from 668 μm to 292 µm; IOP maintained normal at 16 mmHg Case 2. BCVA improved from 20/70 to 20/25; CST decreased from 317 μm to 293 µm; IOP maintained normal at 14 mmHg
Deaner JD, et al. [55]	19	15 M	PSCME after cataract surgery	1. Ten eyes (52.6%) had a ≥ 2-line gain in VA 2. Sixteen eyes (84.2%) had a ≥ 20% reduction in CST. Eight eyes (42.1%) had complete resolution of CME 3. Compared to eighteen eyes (94.7%) requiring local corticosteroid supplementation prior to FAc, only 6 eyes (31.6%) required supplementation after 4. Similarly, of the 12 eyes (63.2%) that were on corticosteroid drops prior to FAc, only 3 (15.8%) required drops after
Patel KG, et al. [56]	24	19.3 M	PSCME after vitrectomy	1. BCVA did not change significantly (*p* = 0.334) 2. CMT improved from 412 µm to a maximum decrease of 311 µm (*p* < 0.001) 3. The injection burden decreased significantly following study treatment (*p* < 0.001) 4. 18 eyes did not require additional intravitreal therapy. 4 eyes requiring intravitreal steroid therapy at median of 7.8 months. One eye never attained sufficient inflammatory control despite rescue therapy
Mahmud et al. [57]	19	6 M	Idiopathic; BRC; MCP; VKH	Uveitis control was achieved in 14 eyes (74%), though three (21%) required a topical steroid after insertion. The remaining five eyes (26%) required additional intraocular treatments
Babel et al. [58]	2	3 M	Idiopathic	1. Follow-up showed improvement in vision, macular edema, and macular leakage on fluorescein angiography imaging: at 3 months after YUTIQ RE and 1 month after YUTIQ LE, BCVA improved from 20/60 to 20/50 RE and 20/70 to 20/40 LE 2. The patient did not have systemic steroid therapy during the course of treatment and IOP remained stable with no IOP elevations following YUTIQ injections OU
Chang PY [59]	4	12-18 M	Idiopathic; Drug induced	1. First patient (OU): In the 18 months following placement of the intravitreal implant, the patient’s inflammation remains quiescent, and visual acuity remained 20/20 bilaterally. The patient’s stage 4 melanoma remained controlled with ongoing immunotherapy. Cataract surgery was performed at 12 months and IOP was controlled with medication 2. Second patient (OU): At the 1- and 2-month follow-ups, vitritis resolved completely, and FA revealed a marked improvement, with central and limited peripheral vascular wall hyperfluorescence still present
Reddy et al. [60]	64	12 M	Idiopathic	1. The overall probability of remaining recurrence-free was 68.8% at six months and 52.6% at 12 months follow-up. Eyes that remained recurrence-free at 12 months had a younger mean age compared to eyes that had a recurrence within 12 months (*p* = 0.02) 2. Eyes that received a short-acting corticosteroid injection prior to YUTIQ were more likely to have a recurrence by six months of follow-up compared to eyes that did not receive a pre-YUTIQ corticosteroid injection (*p* = 0.05) 3. Initiation or addition of IOP lowering eyedrops were required in 15.6% of eyes, and 4.7% of eyes required IOP-lowering surgery following YUTIQ placement

*N* Number of eyes, *VA* Visual acuity, *PSTA* Posterior sub-Tenon triamcinolone, *BCVA* Best corrected visual acuity, *CFT* Central foveal thickness, *IOP* Intraocular pressure, *FAc* Fluocinolone acetonide intravitreal implant, *CRT* Central retinal thickness, *RE* Right eye, *LE* Left eye, *OU* Both eyes, *CME* Cystoid macular edema, *CMT* Central macular thickness, *FA* Fluocinolone acetonide, *ICGA* Indocyanine green angiography, *CS* Corticosteroids, *CVI* Choroidal vascularity index, *CST* Central subfield thickness, *Dex* Dexamethasone intravitreal implant, *BRVA* Best registered visual acuity, *RA* Rheumatoid arthritis, *MS* Multiple sclerosis, *SLE* Systemic lupus erythematosus, *GPA* Granulomatosis with polyangiitis, *PsA* Psoriatic arthritis, *JIA* Juvenile idiopathic arthritis, *BRC* Birdshot retinochoroiditis, *APMPPE* Acute posterior multifocal placoid pigment epitheliopathy, *MCP* Multifocal choroiditis and panuveitis, *AZOOR* Acute zonal occult outer retinopathy, *SC* Serpiginous choroiditis, *BD* Behçet's disease, *SO* Sympathetic ophthalmia, *VKH* Vogt-Koyanagi-Harada, *IRVAN* Idiopathic retinitis, vasculitis, aneurysms, and neuroretinitis, *IU* Infectious uveitis, *PSCME* Post surgical cystoid macular edema, *TINU* Tubulointerstitial nephritis and uveitis, *IRVAN* Idiopathic retinal vasculitis, aneurysms, and neuroretinitis, *AUC* Area under the curve, *Coef* Coeficient, *CMT* Central macular thickness

^a^Summary of diagnosis in NIU-PS treated with fluocinolone acetonide intravitreal implant

^b^Post surgical Cystoid macular edema (PSCME) due to Irvine-Gass syndrome (IGS)

PSME is a primary cause of reduced vision following both cataract and successful vitreoretinal surgery, whose incidence following modern cataract surgery ranged between 0.1 and 3.4% [61, 62]. Although mostly self-limiting, persisting cases can pose a major therapeutic challenge to ophthalmologists, and can mean an increased burden for healthcare systems [63]. Additionally, persistent PSME is often referred to uveitis specialists when the condition has become chronic and manifests as recurrent intraocular inflammation [52].

Although there is no unanimous agreement on the fact of considering relapsing PSME as a uveitis, inflammation plays a key role in its development [61, 62]. Despite this lack of agreement, the panel recommended the use of the FAc implant in these patients (90% agreement) but only in relapsing and chronic cases.

According to the results obtained after the second round of the survey, "strong consensus" (> 95% of the participants agree) or consensus (> 75% to ≤ 95% of the participants agree) was obtained on most of the points addressed.

The panel did not reach consensus (≤ 50% of the participants agree) in two statements:

1. There is no evidence supporting the use of FAc implant as preferable treatment in eyes with inflammatory choroidal neovascularization; therefore, only 50% of the panel recommended its use in these cases.

The second statement on which the panel members did not reach a consensus was the use of the FAc implant, either as monotherapy or as adjunctive therapy, in patients with Tubulointerstitial nephritis and uveitis (TINU). Although there is no prospective, randomized clinical report regarding the treatment of TINU syndrome in the literature, the uveitis in TINU syndrome responds well to topical or systemic steroids in most of the cases [64–66]. However, the disease tends to recur and a slower tapering and long-term treatment with systemic corticosteroids is required [64, 65].

Despite this, the lack of direct evidence evaluating the effectiveness of FAc implant in patients with TINU could be the reason that motivated the lack of a panel consensus.

### Safety: intraocular pressure/cataract

Intravitreal corticosteroid implants may increase the risk of elevated intraocular pressure and cataract formation [67].

Jaffe et al. reported that in eyes with NIU-PS who received a 0.2 µg/day FAc implant, mean intraocular pressure increased by 1.3 ± 3.57 mmHg at month-12 [68]. However, at month-36, the change from baseline was only 0.8 ± 5.0 mmHg and was lower than that observed in the sham-treated group where mean intraocular pressure increased by 1.4 ± 5.7 mmHg [15]. This finding may be related to the fact that in the sham-treated group, many eyes received other systemic and/or topical corticosteroids as a standard of care [15].

In addition to raised intraocular pressure, development of cataract is one of the major concerns when using intravitreal corticosteroids [10, 15, 67–69]. According to the results of the clinical trials, cataract surgery was more frequently required in the FAc implant treated group than in the sham-treated group (73.8% vs. 23.8% of eyes, respectively) [15, 68]. Regarding cataract surgery outcomes, the effect on visual acuity was similar in the FAc implant and sham groups (+ 20.3 letters for the FAc implant-treated group vs. + 23.4 letters for the sham-treated group) [15].

In a post-hoc analysis of a phase-3 randomized clinical trial [15], Pavesio and Heinz [24] compared the clinical outcomes of eyes treated with FAc implant with those of the fellow eye receiving conventional treatments. According to their findings, cataract surgery was more frequently required over 36 months in the FAc implant treated eyes (72%) than in the fellow eyes (37.0%) [24].

### Lens status (Phakic/Pseudophakic/aphakic eyes)

Regarding the use of the FAc implant depending on the state of the lens, the panel showed a strong agreement on the use of FAc implant in pseudophakic eyes (its use is recommended) and in aphakic eyes (its use is not recommended due to the risk of anterior chamber migration, although FAc implant could be considered if sutured to the sclera [51, 53].

With regards to phakic eyes, the panel agreed to recommend the use of the FAc implant. However, the age of the patient, uveitis severity, and the individual limitations for an adequate systemic immunomodulatory therapy need to be considered. Additionally, the use of the FAc implant would be considered in the presence of presbyopia, cataract, or when the patient is undergoing cataract surgery after the implant has been administered.

### Quiescent eye/previous steroid

In order to control active intraocular inflammation in patients with NIU-PS, the panel agreed that DEX-i would be considered as a first-line therapy. This is intended to determine the functionality of corticosteroids, evaluate the incidence of adverse effects (e.g., elevation of intraocular pressure) and whether NIU-PS recurs.

However, it has been published that injecting a DEX-i prior to a FAc implant did not provide better outcomes than inject a FAc implant as first choice [29]. Moreover, there was consensus on the use of the FAc implant in the control of NIU-PS recurrence, when inflammation reoccurs after 1–2 successive DEX-i. In fact, Kessler et al. [27] found that the more corticosteroids administered prior to the FAc implant, the greater the need for combination therapy after FAc implant.

As far as we know, there are no studies comparing FAc implant and repeated injections of DEX-i in patients with NIU-PS. However, it should be highlighted that these implants have been licensed for different indications [12, 70]. While DEX-i was marketed for treating active inflammation, FAc implant is intended to be used to prevent relapses in recurrent NIU-PS. Moreover, the long-lasting effect of FAc implant compared to DEX-i makes FAc implant more effective in the long-term prevention of relapses in recurrent NIU-PS [37], with a significant decrease in the number of intravitreal injections and this represents a reduction in disease burden to the patient.

### Systemic therapies

Current evidence suggests that the 0.2 µg/day FAc implant is effective in reducing the need for subsequent treatment with systemic medication [14, 15, 19, 24, 25, 58, 68, 71, 72].

According to the results of the two pivotal phase 3 randomized, clinical trials, the eyes treated with the 0.2 µg/day FAc implant required fewer adjunctive local and systemic treatments than the eyes treated with sham + standard of care [15, 68, 71].

Jaffe et al. [68] reported that throughout the first 12 months after treatment, the proportions of eyes requiring at least 1 systemic corticosteroids or immunosuppressant treatment was lower in the 0.2 µg/day FAc implant group than in the sham + standard of care treated group (19% versus 40%, respectively).

Throughout the 36-month follow-up period, the proportion of eyes receiving any adjunctive medication in the 0.2 µg/day FAc implant group was 57.5% compared with 97.6% in the sham + standard of care treated group. Moreover, the mean number of adjunctive treatments per eye in the 0.2 µg/day FAc implant group was 0.48 compared with 1.52 in the sham + standard of care treated group [15].

Finally, the results of a retrospective study, conducted on 103 eyes with NIU-PS who underwent treatment with the 0.2 µg/day FAc implant and were followed-up for at least 12 months, found that 55% of patients on oral prednisone and 35% of patients on systemic immunomodulatory therapy at baseline were able to discontinue the therapy by month 12 [72].

### Limitations

It is important to highlight important limitations in this study. Firstly, the clinical experience in some etiologies associated with NIU-PS was limited, and in some cases, absent, which may impact clinical recommendation in these cases. Nevertheless, the broad clinical experience of the panel members, as well as the available evidence with other etiologies might reduce this limitation. In addition, all consensus documents should be considered within an evolving environment and should be regularly revised to implement novel findings as they occur and future evidence as it becomes available.

## Conclusions

According to the panel recommendations, there was agreement that the FAc implant can be considered for use in patients with unilateral, bilateral asymmetrical, and bilateral symmetrical NIU-PS. FAc implant would be used in pseudophakic NIU-PS eyes, but not in aphakic eyes. Regarding the use of the FAc implant in phakic eyes with NIU-PS, the age of the patient, uveitis severity, and the individual limitations for an adequate systemic immunomodulatory therapy need to be considered.

With regards to the use of the FAc implant based on the etiology, its use was recommended as adjunctive/combination therapy in birdshot retinochoroiditis, multifocal choroiditis and panuveitis, serpiginous choroiditis, sarcoidosis, and pars planitis, among others. While its use was not recommended in multiple evanescent white dot syndrome, and acute retinal pigment epitheliitis, due to their transient /self-limiting nature.

This consensus highlights relevant points that may help specialists optimize outcomes in patients with NIU-PS. Moreover, it could serve as a basis to standardize approaches to the management of patients with NIU-PS and to achieve the best outcomes for the patient.

## Supplementary Information

Supplementary Materials 1. Table S1.

Supplementary Materials 2. Annex I.

## Data Availability

Not relevant.

## Abbreviations

AEs: Adverse events
BCVA: Best corrected visual acuity
FAc: Fluocinolone-acetonide sustained-release-0.2 µg/day intravitreal
NIU-PS: Non-infectious uveitis affecting the posterior segment
PSME: Post-surgical macular edema
TINU: Tubulointerstitial nephritis and uveitis
DEX-i: Dexamethasone implant
