# Correction: Fluocinolone acetonide 0.2 µg/day intravitreal implant in non-infectious uveitis affecting the posterior segment: EU expert user panel consensus-based clinical recommendations

**DOI:** 10.1186/s12348-024-00450-w

**Published:** 2025-01-08

**Authors:** Uwe Pleyer, Carlos Pavesio, Elisabetta Miserocchi, Carsten Heinz, Helen Devonport, Víctor Llorenç, Tomás Burke, Vanda Nogueira, Laurent Kodjikian, Bahram Bodaghi

**Affiliations:** 1 Charité – Universitätsmedizin Berlin, Augustenburger Platz 1, 13353 Berlin, Germany; 2 Department of Ophthalmology at Moorfields Eye Hospital, London, UK; 3 Department of Ophthalmology, Ocular Immunology and Uveitis Service, University Vita-Salute, IRCCS San Raffaele Scientific Institute, Milan, Italy; 4 Department of Ophthalmology at St, Franziskus Hospital Muenster, Münster, Germany; 5 Department of Ophthalmology, University Duisburg-Essen, Essen, Germany; 6 Bradford Teaching Hospitals NHS Foundation Trust, Bradford, West Yorkshire UK; 7 Clínic Hospital of Barcelona, Barcelona, Spain; 8 August Pi I Sunyer Biomedical Research Institute (IDIBAPS), Barcelona, Spain; 9 Department of Ophthalmology, Mater Misericordiae University Hospital, Dublin, Ireland; 10 Instituto de Oftalmologia Dr. Gama Pinto, Lisbon, Portugal; 11 Service d’Ophtalmologie, Hôpital Universitaire de La Croix-Rousse, Hospices Civils de Lyon, Lyon, 69004 France; 12 UMR5510 MATEIS, CNRS, INSA Lyon, Université Lyon 1, Villeurbanne, 69100 France; 13 Department of Ophthalmology, Pitié-Salpêtrière Hospital, Paris, France

**Journal of Ophthalmic Inflammation and Infection (2024) 14:22**

10.1186/s12348-024-00402-4

Following publication of the original article, it came to the journal’s attention that ‘Annex I’ (referred to in the Methods subsection ‘Questionnaire development’) was missing from the article. The file has since been added. The authors thank you for reading this erratum and apologize for any inconvenience caused.

