# Supplementary material for: Fluocinolone acetonide 0.2 µg/day intravitreal implant in non-infectious uveitis affecting the posterior segment: EU expert user panel consensus-based clinical recommendations
Source: J Ophthalmic Inflamm Infect. 2024 May 30;14:22. doi: 10.1186/s12348-024-00402-4 (PMC11139823; doi:10.1186/s12348-024-00402-4)
Supplement: Supplementary file 1 — Supplementary Materials 1. Table S1. [file 12348_2024_402_MOESM1_ESM.docx]

Table S1. Classification of the agreement in the consensus decision-making process. Adapted from the German Association of the Scientific Medical Societies (AWMF) – Standing Guidelines Commission [30].

| **Level of Agreement** | **Extent of agreement as a percentage** |
| --- | --- |
| Strong agreement | > 95% of the participants agree |
| Agreement | > 75% ≤ 95% of the participants agree |
| Majority agreement | > 50% ≤ 75% of the participants agree |
| No agreement | ≤ 50% of the participants agree |
