# Supplementary material for: Fluocinolone acetonide 0.2 µg/day intravitreal implant in non-infectious uveitis affecting the posterior segment: EU expert user panel consensus-based clinical recommendations
Source: J Ophthalmic Inflamm Infect. 2024 May 30;14:22. doi: 10.1186/s12348-024-00402-4 (PMC11139823; doi:10.1186/s12348-024-00402-4)
Supplement: Supplementary file 2 — Supplementary Materials 2. Annex I. [file 12348_2024_402_MOESM2_ESM.docx]

Annex I. Questions collected in the survey form.

| **Item** | **Question** |
| --- | --- |
| 1  2  3  4  5  6  7  8  9  10  11  12  13  14  15  16  17  18  19  20  21  22 | Would you use an intravitreal fluocinolone acetonide implant (190 µg) in unilateral uveitis?  Would you use an intravitreal fluocinolone acetonide implant (190 µg) in bilateral but asymmetrical uveitis?  Would you use an that the fluocinolone acetonide intravitreal implant (190 µg) in bilateral symmetrical uveitis?  Is it likely that the fluocinolone acetonide intravitreal implant (190 µg) would be chosen in uveitis with no systemic involvement?  Is it likely that the fluocinolone acetonide intravitreal implant (190 µg) would be chosen in uveitis in association with systemic involvement?  Is it likely that the fluocinolone acetonide intravitreal implant (190 µg) would be chosen in phakic eyes?  Is it likely the fluocinolone acetonide intravitreal implant (190 µg) would be chosen in pseudophakic eyes?  Is it likely the fluocinolone acetonide intravitreal implant (190 µg) would be chosen in aphakic eyes?  Would the fluocinolone acetonide intravitreal implant (190 µg) be chosen as a treatment method for any non-infectious uveitis with or without macular edema?  Would the fluocinolone acetonide intravitreal implant (190 µg) be chosen as a treatment method preferentially for non-infectious with macular edema?  Would the fluocinolone acetonide intravitreal implant (190 µg) be chosen as a treatment method only for non-infectious uveitis with macular edema?  Assuming a patient is already on systemic immunomodulation, what would your treatment approach be for a patient with recurrent active intraocular inflammation?  Would you consider treating locally to prevent recurrence of intraocular inflammation?  Regardless of systemic treatment, would the presence/absence of choroidal neovascularization impact your decision to use local corticosteroids?  Regardless of systemic treatment, would the presence/absence of primordial choroidal inflammation impact your decision to use local corticosteroids?  Which local therapy would you use first in your personal treatment scheme?  Which situation would you consider the eye to be quiescent?  When would you inject the fluocinolone acetonide intravitreal implant (190 µg)?  Would the fluocinolone acetonide intravitreal implant (190 µg) be considered as an acceptable adjunctive treatment or in the prevention of recurrences in non-infectious uveitis?  Would the fluocinolone acetonide intravitreal implant (190 µg) be considered as an acceptable monotherapy treatment or in the prevention of recurrences in non-infectious uveitis?  Would the re-injection of the fluocinolone acetonide intravitreal implant (190 µg) be considered in the prevention of recurrence in non-infectious uveitis?  What would be the reason(s) to re-inject the fluocinolone acetonide intravitreal implant (190 µg) in the prevention of recurrence in non-infectious uveitis? |
